# How do cultural repertoires shape sustainable teacher identity? Confucian striving and Daoist release among Chinese pre-service teachers

**DOI:** 10.3389/fpsyg.2026.1986498

**Published:** 2026-10-15

**Authors:** Xuefei Lin, Qi Tan, Bin Xiong

**Affiliations:** 1 School of Mathematics, Yangzhou University, Yangzhou, China; 2 Shanghai Key Laboratory of Pure Mathematics and Mathematical Practice, Shanghai, China; 3 School of Mathematics and Statistics, Hunan Normal University, Changsha, China; 4 School of Mathematical Sciences, East China Normal University, Shanghai, China

**Keywords:** Confucianism, cultural repertoires, Daoism, pre-service teachers, teacher identity

## Abstract

**Introduction:**

Teacher identity formation is a dynamic process shaped by professional experience, emotion, wellbeing, and culturally available meanings. This study examines how pre-service teachers in China drew on Confucian and Daoist repertoires when reflecting on their developing teacher identities.

**Methods:**

Participants were 143 pre-service teachers at one Chinese normal university. They wrote prompted reflective narratives about coursework, micro-teaching, lesson planning, educational observation, and imagined classroom responsibilities. The analysis combined descriptive keyword mapping with reflexive thematic analysis.

**Results:**

Four related processes were identified: Confucian striving as a language of perseverance and self-cultivation; Daoist release as a resource for non-contention, recognizing limits, and emotional restoration; situational switching between these repertoires in response to changing demands; and sustainable teacher formation as an orientation toward responsibility with limits. The study proposes cultural identity regulation as an integrative analytic heuristic for understanding how culturally available meanings connect academic experiences, emotional calibration, and imagined teacher selves.

**Discussion:**

The findings suggest that Confucian striving and Daoist release provided participants with culturally familiar ways to interpret effort, pressure, and anticipated responsibility during teacher preparation. Their value lay in helping participants connect perseverance with self-cultivation and responsibility, while also recognizing limits, reducing destructive comparison, and restoring emotional balance. Cultural identity regulation captures this narrated negotiation of professional commitment and personal sustainability in the formation of an imagined teacher self.

## Introduction

Teacher education is not only a process of acquiring pedagogical knowledge, instructional skills, and classroom routines. It is also a process through which pre-service teachers learn how to read feedback, handle uncertainty, prepare lessons, imagine pupils, and decide what kind of teacher they can become. Contemporary teacher identity research conceptualizes identity as relational, emotional, narrative, and situated (Akkerman and Meijer, 2011; Beauchamp and Thomas, 2009; Rushton et al., 2023). This process begins before formal coursework, since students enter teacher education with memories of schooling and assumptions about good teaching (Lortie, 1975). Teaching is also moral work because it involves care, judgment, and responsibility toward learners (Campbell, 2008). The present study asks how culturally familiar repertoires enter this process in one mathematics education course, before extended classroom practice, when students are still forming the habits with which they will later encounter practicum.

Existing research has demonstrated that teacher identity develops through interactions among biography, institutional expectations, emotions, recognition, and vulnerability (Beijaard et al., 2004; Kelchtermans, 2009). Recent work further shows that identity remains active across teachers’ professional lives and is shaped by agency, tensions, and wellbeing (Groenewald and Arnold, 2024; Rushton et al., 2023). Less attention has been paid to the culturally inherited interpretive resources through which pre-service teachers make sense of challenges, interpret feedback, and regulate the early development of professional identity.

This question is particularly salient in Chinese teacher education, where teaching has long been associated with moral cultivation, self-discipline, exemplarity, and social responsibility. Contemporary Chinese pre-service teachers also face academic competition, credential pressure, performance evaluation, and uncertain career prospects (Lin et al., 2021). Confucian sayings can be used to frame perseverance and responsibility toward future students, whereas Daoist sayings can be used to frame acceptance, proportion, emotional restoration, and freedom from excessive comparison (Chen, 2010). The study does not assume that either tradition simply produces pressure or wellbeing; rather, it examines how these resources were actually used within a specific prompted writing activity.

Against this background, the study examines how pre-service teachers at one Chinese normal university mobilized Confucian and Daoist cultural repertoires when narrating academic difficulty, teaching preparation, micro-teaching, educational observation, and imagined classroom responsibility. We develop cultural identity regulation as an integrative analytic heuristic. In this study, cultural identity regulation refers to the narrated use of culturally available sayings to interpret professional experiences, evaluate competing demands, and connect responses such as persistence or recovery with an imagined future teacher self. This is a claim about narrative practice, not about an internal psychological trait. The analysis is guided by three questions:

RQ1: How did participants use culturally inherited repertoires to regulate professional identity demands within the prompted reflections?

RQ2: How did situational movement between striving and release appear in participants’ anticipatory accounts of sustainable professional becoming?

RQ3: What analytic insights does the cultural identity-regulation heuristic offer for teacher identity theory, wellbeing, and teacher education beyond the Chinese case?

## Literature review

### Teacher identity as situated professional becoming

Contemporary teacher identity research has moved beyond the assumption that becoming a teacher simply involves acquiring pedagogical knowledge or adopting a professional role. Identity is now understood as situated, relational, and dynamic, constructed through personal histories, institutional expectations, professional relationships, and imagined futures (Sfard and Prusak, 2005; Wenger, 1998). Because teachers are evaluated for the kinds of professionals they are expected to become, identity formation is also vulnerable and emotionally demanding (Kelchtermans, 2009).

Research has increasingly emphasized that teacher identity develops through interactions among personal meaning, social recognition, institutional positioning, and professional agency. Identity is simultaneously an individual and social achievement because it depends on how teachers understand themselves and how they become recognizable within educational communities (Gee, 2000). Dialogical perspectives conceptualize teacher identity as involving multiple and sometimes competing self-positions that shift across situations and relationships (Akkerman and Meijer, 2011). Narrative approaches likewise demonstrate that teachers organize professional becoming through stories connecting past experience, present practice, and imagined futures (Beauchamp and Thomas, 2009). Recent studies of identity tensions further show that becoming a teacher involves negotiating competing values rather than simply consolidating one stable role (Hanna et al., 2022; Nickel and Crosby, 2022). Identity formation is therefore best understood as a process through which individuals negotiate professional possibilities within particular social, institutional, and cultural contexts.

Context has become central in teacher identity scholarship. Flores and Day (2006) show that professional identities emerge through the interaction of personal histories and institutional environments. Yet discussions of context often focus on programs, practicum, school cultures, policy, and mentoring. Less attention has been paid to the culturally inherited interpretive resources through which pre-service teachers make sense of those experiences.

The study addresses this cultural dimension by drawing on Swidler's (1986) conception of culture as a repertoire of symbols, stories, and strategies for action. Rather than treating culture as a fixed system that determines behavior, this perspective emphasizes how individuals selectively mobilize available resources to interpret experience. Narrative theories similarly show that identity is organized through culturally available storylines connecting past events with future aspirations (Bruner et al., 1993; McAdams, 2001). Cultural worlds also provide recognizable positions and forms of agency through which people learn who they can become (Holland et al., 1998).

Building on these perspectives, the study treats Confucian and Daoist sayings as identity resources through which Chinese pre-service teachers interpret effort, responsibility, emotional strain, self-limitation, and future teacherhood. Participants used classical sayings to connect remembered learning experiences, present identity work, and imagined teacher selves. Teacher identity is therefore understood as situated, culturally storied, and shaped through institutional experience, narrative meaning-making, and inherited repertoires.

### Wellbeing, resilience, and sustainable professional becoming

Teacher identity formation is closely intertwined with wellbeing and resilience. Contemporary teacher education encourages commitment, care, and professional responsibility, yet these ideals become fragile when detached from emotional support and realistic professional limits. Research on teacher resilience shows that thriving depends on the interaction between personal resources and professional contexts (Beltman et al., 2011; Gu and Day, 2007). Teacher wellbeing is likewise understood as a multidimensional and consequential feature of educational life, not merely the absence of stress (Collie et al., 2015; Dreer, 2023; Hascher and Waber, 2021). For pre-service teachers, wellbeing is also linked to identity, emotion regulation, and the capacity to remain professionally engaged while learning to manage pressure (Hui et al., 2024; Squires et al., 2022).

These perspectives are particularly relevant during pre-service teacher education, when students are still constructing images of what it means to become a good teacher. Many enter teacher education with idealized expectations of teachers as endlessly patient, knowledgeable, morally exemplary, and self-sacrificing. Such ideals can strengthen commitment, but they may also encourage self-blame, emotional exhaustion, and unrealistic standards when ordinary difficulties arise. Recent studies of transition into teaching similarly show that early professional identity is shaped through tensions among values, resilience, agency, and vulnerability (Cobb, 2022; Nickel and Crosby, 2022). Sustainable teacher formation therefore does not imply reduced commitment. It refers to professional becoming in which commitment can be maintained without chronic self-sacrifice or destructive comparison.

The present study contributes to this literature by suggesting that resilience is supported not only by personal skills or institutional resources but also by culturally meaningful ways of interpreting professional experience. The participants’ narratives indicate that Confucian striving and Daoist release functioned as complementary interpretive resources through which they negotiated perseverance, emotional recovery, responsibility, and professional sustainability. In this sense, wellbeing is understood not merely as an individual psychological outcome but as an ongoing process of identity regulation during teacher preparation. Recent work also connects pre-service teacher identity with wellbeing and emotion regulation, suggesting that identity support should be treated as part of wellbeing education rather than as a separate professional issue (Hui et al., 2024).

### Chinese teacher education and cultural repertoires

Chinese teacher education provides an important context because professional preparation has long been associated with moral cultivation, social responsibility, and ethical self-development. Chinese pre-service teachers actively negotiate teacher morality, civic responsibility, care, authority, and ethical role construction rather than simply accepting institutional expectations (Ye and Law, 2019; Ye et al., 2022). Traditional cultural ideas also shape understandings of science, teaching values, and professional motivation (Sang, 2023; Liang et al., 2023). Recent work shows that Chinese teacher identity is negotiated through institutional, social, and cultural positioning (Liang et al., 2023). Early professional identity is also relevant to retention and commitment (Hong, 2010), while recent profile-based work shows that pre-service teacher identities are differentiated and responsive to teacher education contexts (Meyer et al., 2023).

These findings indicate that Confucianism and Daoism continue to influence teacher education, not primarily as formal religious systems but as culturally available repertoires for interpreting professional life. Confucian traditions are associated with learning, self-cultivation, moral exemplarity, and relational responsibility, whereas Daoist traditions emphasize naturalness, non-contention, restraint, and the avoidance of excessive control. Research on East Asian belief systems cautions against treating these traditions as mutually exclusive categories (Rosenfeld et al., 2022).

What’s more, an important conceptual gap remains. Teacher identity research has generated sophisticated accounts of narrative identity, emotion, resilience, and professional becoming, while Chinese teacher education research has examined morality and ethical role construction. Cultural studies have clarified Confucian and Daoist traditions. However, less is known about how inherited repertoires operate within the everyday identity work of pre-service teachers. This study bridges these perspectives by examining how sayings become practical identity resources for interpreting effort, emotional strain, responsibility, and future professional selves.

### Theoretical perspective

The study draws on three complementary bodies of work. Teacher identity theory conceptualizes becoming as ongoing negotiation rather than the acquisition of a fixed role. Dialogical approaches suggest that identity involves multiple self-positions (Hermans, 2001), while teacher identity research emphasizes reconstruction through experience and interaction (Akkerman and Meijer, 2011). Cultural repertoire theory proposes that individuals selectively draw on symbols, stories, and strategies when responding to situations (Swidler, 1986). Taken together, these perspectives justify examining classical sayings as culturally available language through which participants can narrate effort, responsibility, emotional strain, and future teacherhood. They do not, by themselves, prescribe the four findings reported below.

Building on this foundation, the study proposes cultural identity regulation as an analytic heuristic. In this study, cultural identity regulation refers to the narrated use of culturally available sayings to interpret professional experiences, evaluate competing demands, and connect responses such as persistence or recovery with an imagined future teacher self. This general process is operationalized through three mechanisms. Moral orientation names the process through which cultural repertoires define what counts as worthy effort, responsible care, and a good teacher self. Emotional calibration names the process through which students judge whether a situation calls for persistence, reduction, acceptance, or recovery. Sustainable professional agency names the capacity to continue becoming a teacher without converting every difficulty into self-blame or every limit into withdrawal. Within a narrative sequence, the heuristic asks what cultural resource is activated, what moral orientation or emotional calibration it authorizes, and how the response is linked to an imagined teacher self. Confucian striving and Daoist release are therefore not treated as opposing doctrines. They are situational resources through which students regulate identity demands.

This contribution is deliberately modest. The study uses these traditions as conceptual resources rather than treating their combination as a new general theory of identity. The heuristic is developed from the present corpus and is intended to make the cultural content of identity positions analytically visible. It should be assessed in future research with other groups and observational data. The analytic framework is specified in the Method section, where its relation to data generation and coding is made explicit.

The framework advances teacher identity theory by moving from the broad claim that culture shapes identity to a more precise account of identity regulation. It specifies the cultural content of dialogical positions: participants positioned themselves as self-cultivating learners, responsible future teachers, non-contending peers, and professionals who knew when to accept limits. It also shows that future teacher identities are culturally narrated, since present academic struggle became preparation for future professional care. This point connects with work that treats teacher education as negotiation between personal and professional discourses (Alsup, 2006; Walkington, 2005).

Finally, the framework provides a cultural perspective on professional vulnerability. Teacher identity research has long argued that teachers’ self-understanding remains vulnerable because teaching involves continuous evaluation by oneself and by others (Kelchtermans, 2009). This study suggests that cultural repertoires shape how vulnerability is interpreted: failure, competition, or emotional strain may become occasions for self-cultivation, signals to restore balance, evidence of inadequacy, or prompts for reflection. Figure 1 presents the framework.

**Figure 1:**
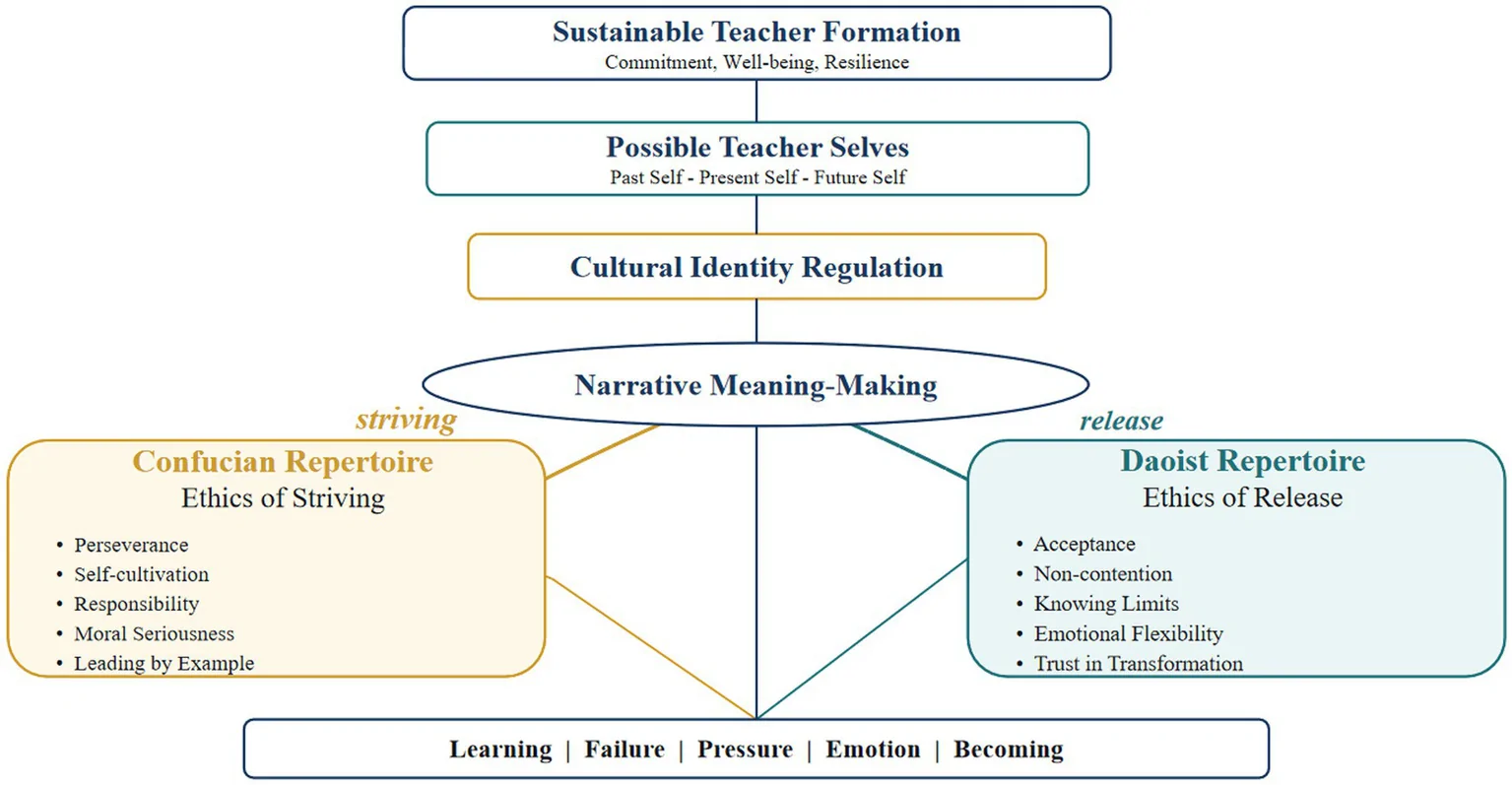
Cultural identity-regulation framework.

## Methodology

### Ethics statement

This study obtained informed consent from participants and approval from the ethics committee of the authors’ institution.

### Research design and participants

This study used corpus-supported reflexive thematic analysis to examine prompted reflective narratives about learning, emotions, and anticipated professional growth. The approach combines close qualitative interpretation with a limited descriptive mapping of recurring language. Reflexive thematic analysis was selected because it supports the identification of patterned meaning while retaining attention to context, researcher interpretation, and ambiguity (Braun and Clarke, 2021; Byrne, 2022).

The dataset comprises 143 prompted reflective narratives produced by pre-service teachers at a century-old teacher education university in Jiangsu Province, China, a highly specialized teacher training institution within the Chinese teacher education system. Participants were preparing to teach either primary general education or secondary mathematics education, and all texts were generated within the context of their mathematics education course.

The writing activity occupied approximately 45 min of scheduled class time and was facilitated by a researcher. Participants were asked to select a classical saying or anecdote associated with either the Confucian or Daoist tradition no fixed list was imposed; they chose the saying they wished to discuss explain its personal significance, and relate it to one or more of the following areas: academic pressure, academic setback, social comparison, emotional experience, moral development, or future teaching practice. After class, the researchers conducted informal interviews with students who volunteered to participate and carried out random sampling checks of the essays to verify authenticity. All interviewees passed the authenticity check; the corpus was therefore treated as authentically authored reflections.

Ethical safeguards were built into the activity in advance. Before the class session, students were informed of the session’s basic content and of potential risks to participants. Participation was entirely voluntary: students who chose not to contribute a text incurred no penalty of any kind, and those who opted out of the writing activity remained welcome to join the subsequent discussion and exchange. Students could also withdraw from the study at any time, without giving a reason.

Demographic information is summarized in Table 1. The sample was approximately gender-balanced. It should be emphasized that the primary data source is a corpus of reflective narratives generated through this prompt-based writing activity, rather than interview data in the conventional sense. Because the archived materials do not include complete classroom recordings or verbatim transcripts, these data are best characterized as reflective identity narratives rather than conversational interview records.

**Table 1:** Sample description (*N* = 143).

Demographic	Type	Frequency	Percentage
Gender	Male	69	48.25%
	Female	74	51.75%
Grade	Grade 1	71	49.65%
	Grade 2	70	48.95%
	Grade 3	2	1.40%
Major	Mathematics and Education	73	51.05%
	Education	70	48.95%

Because the prompt explicitly foregrounded the Confucian and Daoist traditions and linked them to study and teaching, the prominence of these repertoires in the corpus is partly task-elicited. The findings therefore concern how participants used this provided cultural frame in reflective writing, not the spontaneous prevalence of these traditions in participants’ everyday identity work. This delimitation is consistent with the study’s broader claim: the analysis examines the use of culturally available repertoires, not their background frequency (cf. Swidler, 1986).

Finally, the classroom setting created a plausible social-desirability and demand-characteristics risk. Participants knew that the activity formed part of a teacher education course, where diligence, self-cultivation, and responsibility carry positive institutional value. Although the consent procedures described above stressed voluntariness and anonymity, they cannot eliminate the possibility that some narratives were shaped by institutional expectations, the instructor’s presence, or an anticipated audience. This means our results should be viewed with caution.

### Analytical strategy: integrating breadth and depth

Given the size of the corpus, the analysis combined corpus-level mapping with close interpretive reading to balance breadth and depth. Corpus mapping was used to identify the distribution and visibility of recurring cultural and identity-related language across the 143 narratives, while close reading examined how these resources functioned within particular narrative sequences. Particular attention was paid to accounts involving identity tension, ambivalence, emotional change, and movement between different cultural positions. This combined strategy reduced reliance on isolated illustrative quotations while retaining the contextual and interpretive orientation of the analysis. It also aligns with approaches to teacher professional learning that emphasize the interpretation and transformation of experience (Johnson and Golombek, 2018).

The coding framework was organized hierarchically. At the first level, recurring Chinese expressions and related everyday vocabulary were organized into four broad analytic categories: Confucian Striving, Daoist Release, Responsibility and Service, and Identity Regulation. These categories provided a corpus-level map of the cultural and professional discourses visible across the narratives. At the second level, close qualitative coding used 11 operational codes specified in the codebook: Striving/Perseverance, Self-Cultivation, Vulnerable/Moralized Striving, Acceptance/Release, Non-Contention/Comparison Release, Knowing Limits/Emotional Restoration, Avoidance/Maladaptive Release, Teacher Responsibility/Service, Future Teacher Self, Situational Switching, and Sustainable Teacher Formation. Coding was conducted at the level of meaning units rather than isolated words. Multiple codes could be assigned to the same meaning unit when it performed more than one analytically distinct function. Particular attention was given to boundary cases, including the distinction between striving and self-cultivation, adaptive release and avoidance, responsibility and an imagined future teacher self, and situational switching versus the simple co-occurrence of Confucian and Daoist resources.

Corpus frequencies were then used descriptively to establish the visibility of the four broad analytic categories and their associated language. Document-level occurrence and total mentions were calculated and considered alongside the qualitative coding. These frequencies were not treated as evidence of thematic importance in themselves; rather, they provided a corpus-level context for interpreting how cultural resources were used within narratives. The final themes were developed through iterative reading across coded meaning units, with particular attention to the sequence of dilemma, cultural resource, response, and professional implication. This process generated four interpretive themes: Confucian striving, Daoist release, situational switching, and sustainable teacher formation. The keyword distributions are presented in Table 2, while the full hierarchical codebook is provided in Appendix A.

**Table 2:** Keyword frequency by analytic category.

Analytic category	Keyword group	Documents	Frequency
Confucian striving	Study/learning	143	6,274
	Moral cultivation	127	688
	Effort/diligence	128	606
	Difficulty/setback	101	275
	Self-strengthening	79	268
Daoist release	Water/non-contention	46	229
	Calm/acceptance	74	117
	Knowing limits	32	109
	Naturalness	51	88
	Non-action/restraint	45	66
Identity regulation	Growth	135	907
	Balance	107	296
	Reflection	104	283
	Comparison	93	258
	Future orientation	74	143
Responsibility and service	Future teacher	107	575
	Service beyond self	49	132
	Public concern	60	120
	Care/guidance	74	109
	Responsibility	23	33

The subsequent coding cycles moved increasingly from descriptive categorization toward process-oriented interpretation. Rather than treating Confucian and Daoist sayings as stable or mutually exclusive cultural categories, the analysis examined how participants mobilized them in response to particular demands and how their meanings changed within narratives. The analysis therefore followed a recurring sequence of questions: What dilemma or demand was being narrated? Which cultural resource was activated? What behavioral, emotional, or interpretive response followed? How was that response connected to an imagined future teacher self? Cross-case comparison and repeated rereading of coded meaning units ultimately generated four interpretive themes: Confucian striving as difficulty transformed into material for teacher becoming; Daoist release as the negotiation of limits, non-contention, and emotional restoration; situational switching between striving and release; and sustainable teacher formation as responsibility combined with limits and restoration. These themes were treated as interpretive identity processes rather than direct summaries of the operational codes.

### Coding calibration and credibility assurance

To assess consistency in applying the operational codebook, the two researchers independently coded 30 de-identified meaning units. Agreement was high (Cohen’s *κ* = 0.813; observed agreement = 94.8%), indicating strong convergence in the application of the coding framework. Disagreements were subsequently discussed and resolved with reference to the codebook and the surrounding narrative context.

These agreement statistics formed only one component of a broader credibility strategy. Reflective memo writing, audit trails, corpus-level keyword mapping, repeated cross-referencing with the original Chinese texts, peer debriefing, and attention to negative and ambiguous cases were used throughout the analysis. Peer debriefings specifically examined whether the developing interpretation over-romanticized cultural traditions or underestimated stress, avoidance, contradiction, and ambiguity. Negative cases were especially informative. Some participants described Confucian effort orientations as sources of excessive pressure or moralized self-demand, whereas Daoist language of acceptance or naturalness could, when detached from responsibility, become a justification for avoidance. Such cases were retained within the coding framework rather than treated as deviations to be removed.

Consistent with reflexive thematic analysis, the agreement statistics were therefore used as descriptive evidence of coding calibration and convergence in applying the operational codebook, rather than as a test of the objectivity or reliability of the final interpretive themes (Braun and Clarke, 2022; Byrne, 2022). The analysis remained iterative throughout. Early coding treated Confucian and Daoist resources more separately, but repeated comparison across narratives showed that this distinction alone could not explain how participants negotiated pressure, responsibility, recovery, and future teacherhood. The final thematic framework consequently emphasizes the dynamic relationships among cultural resources and the ways in which participants used them to regulate professional identity demands.

## Results

### From analytic categories to identity processes

The analysis moved from four broad analytic categories to four higher-order interpretive themes. The categories of Confucian Striving, Daoist Release, Responsibility and Service, and Identity Regulation organized the corpus and provided the initial structure for meaning-unit coding. The final themes, however, were not intended to reproduce these categories directly. Rather, they captured patterned relationships among the operational codes and showed how cultural resources functioned within participants’ narratives of professional becoming. Confucian Striving and Daoist Release remained recognizable as two major cultural processes, whereas Responsibility and Service became increasingly intertwined with participants’ imagined future teacher selves. Identity Regulation was most clearly expressed through situational movement between striving and release and through attempts to reconcile professional commitment with emotional and personal limits. The resulting four themes were therefore Confucian striving, Daoist release, situational switching, and sustainable teacher formation.

Figure 1 summarizes this analytic progression. Confucian striving was primarily constructed from Striving/Perseverance and Self-Cultivation, while also incorporating Vulnerable/Moralized Striving as an important negative case. Daoist release drew primarily on Acceptance/Release, Non-Contention/Comparison Release, and Knowing Limits/Emotional Restoration, with Avoidance/Maladaptive Release marking its principal boundary condition. Situational switching emerged from narratives in which participants moved between these repertoires according to changing demands rather than simply mentioning both traditions. Sustainable teacher formation brought together Teacher Responsibility/Service, Future Teacher Self, and Sustainable Teacher Formation codes, often through narratives in which commitment was recalibrated by limits, restoration, and situational judgment. Thus, the themes represent higher-order identity processes developed from relationships among codes rather than one-to-one restatements of the coding categories.

### Confucian striving: from perseverance to self-cultivation

Participants most often mobilized Confucian sayings when narrating academic difficulty, sustained effort, self-correction, and the moral seriousness of preparing to teach. Across these accounts, however, striving involved more than persistence. The qualitative coding distinguished between Striving/Perseverance, in which cultural language supported continued action in the face of difficulty, and Self-Cultivation, in which difficulty itself was reinterpreted as material for developing character and a professional self. The distinction was important because many narratives moved from the question of whether to continue toward the question of what kind of person, and eventually what kind of teacher, one might become through continuing.

One participant described this shift after repeated academic setbacks:

*After several failed quizzes, I stopped thinking that difficulty meant I was not suited to mathematics. I began to see difficulty as the place where I should cultivate myself.*

Here, failure was no longer narrated solely as evidence of mathematical inadequacy. It was repositioned as an occasion for self-cultivation. A related but more action-oriented account emphasized perseverance:

*Self-strengthening made me divide the large task into today’s small task. Only after doing that did I feel that I could continue.*

The distinction between these accounts illustrates the relationship between two operational codes. In the second account, self-strengthening primarily authorized continued action; in the first, difficulty acquired a broader meaning for the formation of the self. Across the corpus, these functions frequently overlapped, but they were analytically distinguishable: perseverance concerned how participants continued, whereas self-cultivation concerned how they interpreted what continuing meant for who they were becoming.

This movement from effort toward self-formation also appeared in relational situations. Participants drew on Confucian ideas of self-reflection and virtue when describing disagreement in group work, often turning attention away from blaming others and toward their own conduct. One participant reflected:

*Before blaming others, I had to ask whether I had really listened, explained clearly, and taken responsibility for my own part.*

Such accounts extended self-cultivation beyond individual achievement. Reflection on one’s own shortcomings became connected with listening, cooperation, and responsibility toward others. In this sense, Confucian striving functioned not only as a language of persistence but also as a form of relational self-correction through which participants began to position themselves in relation to the responsibilities associated with teaching.

The connection became more explicit in narratives about lesson planning, micro-teaching, and educational observation. One participant initially approached lesson planning by reproducing textbook and online materials but reconsidered this strategy after trial teaching revealed weak alignment with students’ understanding:

*When preparing a teaching design for educational observation, I first copied textbook and online lesson plans. During trial teaching I found that the design did not fit students’ understanding, and classroom interaction was weak. I returned to the saying about learning widely, asking carefully, thinking cautiously, distinguishing clearly, and practicing earnestly. I read curriculum standards, asked my mentor about students’ common mistakes, revised the examples, compared different lesson structures, and practiced again.*

In this narrative, the cultural resource did not merely encourage the participant to “work harder.” It organized a sequence of professional action: identifying inadequacy, seeking information, consulting a mentor, reconsidering pupils’ understanding, revising the lesson, and practicing again. Confucian striving thus became connected with the concrete work of learning to teach.

A similar movement from self-development toward professional responsibility appeared in a narrative drawing on a Mencian saying:

*At the beginning of university I felt uncertain about the future of the teacher education major. I did not know how to prepare for teaching practice, so I first focused on cultivating myself. I completed my professional courses carefully, listened to open classes given by experienced teachers, repeatedly polished my micro-teaching script, and learned educational psychology and speaking skills through student activities. During the second-year educational observation, I could help the mentor teacher mark homework and discuss lesson design. The saying helped me understand that self-cultivation is the basis for later service.*

This account illustrates how Self-Cultivation became connected with the broader analytic category of Responsibility and Service. Professional preparation was narrated as a movement from improving the self toward becoming capable of contributing to others. Rather than treating academic learning and professional identity as separate domains, participants sometimes used culturally familiar language to connect present learning practices with anticipated responsibilities toward future pupils.

This connection was especially visible when participants explicitly projected current experiences into an imagined future teacher self. As one future mathematics teacher wrote:

*I hope to pass perseverance to my students, but not the pressure that once made me afraid of every mistake.*

The statement contains both continuity and revision. Perseverance remained a valued resource, but the participant did not simply intend to reproduce the form in which it had previously been experienced. Instead, past pressure became a basis for imagining a different pedagogical relationship with future students. In coding terms, Striving/Perseverance and Future Teacher Self intersected with Teacher Responsibility/Service: a personal learning experience was reconstructed as a prospective principle for guiding pupils.

Importantly, the Confucian repertoire was not uniformly experienced as supportive. A smaller set of accounts indicated that self-strengthening could become Vulnerable/Moralized Striving when persistence was interpreted as an obligation to continue regardless of exhaustion or personal limits. These negative cases complicate any reading of Confucian striving as inherently adaptive. They suggest instead that its identity significance depended on how effort was interpreted and what kind of professional self it authorized. This boundary becomes particularly important in the later themes, where participants drew on Daoist resources to distinguish sustained commitment from excessive self-demand.

Taken together, these narratives show a recurring progression from perseverance to self-cultivation and, in some cases, from self-cultivation to imagined professional responsibility. Confucian sayings helped participants narrate academic difficulty as something that could be acted upon, interpreted, and incorporated into a developing teacher self. Yet the same repertoire also contained a potential tension: the moral seriousness that supported persistence could become burdensome when effort was detached from limits. This tension provides an important transition to the second theme, in which Daoist resources offered a different way of interpreting pressure, comparison, and the need for emotional restoration.

### Daoist release: from non-contention to emotional restoration

Daoist sayings were most often mobilized when participants narrated overload, social comparison, anxiety, or a perceived need to regain proportion. In contrast to Confucian striving, which frequently authorized continued effort and self-cultivation, Daoist resources tended to change participants’ relationship to effort itself. The operational coding distinguished among Acceptance/Release, Non-Contention/Comparison Release, and Knowing Limits/Emotional Restoration. Across these codes, release rarely meant simply withdrawing from learning. More often, participants used Daoist language to distinguish productive engagement from forms of effort, comparison, or control that they had come to regard as excessive or unhelpful.

This distinction was particularly visible in narratives about academic overload. One participant described accumulating exercise books and solving problems mechanically every day, only to experience increasing confusion. The saying For learning one increases daily; for the Way one decreases daily provided a different interpretive position from which the participant could reduce rather than intensify effort:

*I was doing more and more exercises, but my heart became more and more chaotic. Reducing was not laziness; it was the beginning of understanding.*

Another participant similarly wrote:

*When I stopped collecting materials and returned to the key ideas, I felt that learning finally had a center again.*

These accounts illustrate Acceptance/Release as selective reduction rather than disengagement. The cultural resource made it possible to reinterpret “doing less” as a deliberate response to ineffective effort. What changed was therefore not simply the amount of work but the meaning attached to reduction: stopping, narrowing, or slowing down could be narrated as a way of restoring direction rather than as evidence of insufficient commitment.

A related but analytically distinct pattern appeared in narratives of ranking and social comparison. Here, Non-Contention/Comparison Release involved loosening the connection between external comparison and self-evaluation. One participant described discomfort after a classmate received a higher examination score despite the participant’s own intensive preparation. Reflection on the image of water benefiting all things without contending did not lead the participant to reject achievement. Instead, it provided a way of questioning whether a single comparison should determine personal worth:

*Non-contention did not mean giving up. It meant not letting one score decide my worth.*

Another participant described the emotional consequence of temporarily stepping outside comparison:

*When I stopped comparing for a while, I could breathe and study again.*

*The distinction is important. Non-contention did not necessarily reduce participants’ commitment to learning; rather, it could reposition the basis on which that commitment was evaluated. Attention shifted from outperforming others toward personal progress, while temporary distance from comparison created what participants described as greater emotional space. In this sense, non-contention intersected with Knowing Limits/Emotional Restoration: release from comparison was narrated not as an endpoint but as a way of recovering sufficient proportion to re-engage with learning.*

These narratives should not be read as evidence that Daoist resources produced measurable improvements in wellbeing. Rather, they show how culturally familiar language made acceptance, selectivity, and emotional recovery narratively legitimate within the prompted reflections. Participants could describe stepping back without necessarily construing that response as weakness or failure. In several accounts, knowing when to stop, reduce, or accept limits became part of how participants imagined sustaining engagement under pressure.

Some narratives extended this reasoning from personal learning to anticipated teaching. Participants described future teaching in terms of creating appropriate conditions for learning, respecting differences in student pace, and recognizing situations in which patience might be more appropriate than tighter control. These accounts connected Daoist Release with the Future Teacher Self, although they remain anticipatory professional orientations rather than evidence of enacted classroom practice. The significance lies in how participants used present experiences of pressure and recovery to imagine alternative ways of responding to future pupils.

At the same time, the coding revealed an important boundary between adaptive release and Avoidance/Maladaptive Release. Daoist language was interpreted as release when reducing effort or accepting limits retained a reflective purpose and preserved meaningful engagement. The same language could become problematic when “letting go” functioned primarily as a justification for evading difficult but necessary action. As one participant put it:

*Letting go is not doing nothing. It is knowing which pressure is useful and which pressure only makes me smaller.*

The distinction between release and avoidance prevents Daoist resources from being interpreted as inherently restorative. Their meaning depended on what followed from them: reduction could restore clarity, non-contention could loosen destructive comparison, and recognition of limits could create emotional space, but disengagement remained a possibility when release became detached from responsibility. This ambivalence parallels the boundary identified in the previous theme, where Confucian striving could support perseverance but could also become moralized self-demand.

Taken together, these narratives suggest a progression from release from unproductive effort, to release from comparison, to recognition of limits and emotional restoration. Yet neither striving nor release appeared sufficient on its own. Participants’ accounts increasingly raised a situational question: when did a difficulty require renewed effort, and when did continued effort itself become part of the problem? This question provides the transition to the third theme, situational switching, in which identity regulation became visible through movement between culturally available positions rather than through stable adherence to either repertoire.

### Situational switching: identity regulation across repertoires

A central analytic pattern emerged not from Confucian striving or Daoist release alone, but from the narrated movement between them. Whereas the first two themes showed how particular repertoires could authorize persistence, self-cultivation, reduction, or emotional restoration, participants also described situations in which the appropriateness of these responses depended on the demands of the moment. In the operational coding, Situational Switching captured this movement: it was assigned when participants explicitly contrasted, repositioned, or conditionally selected different cultural responses rather than merely mentioning Confucian and Daoist ideas within the same narrative. In this sense, situational switching represented one of the clearest narrative expressions of Identity Regulation.

Participants commonly associated Confucian language with moments requiring renewed effort, discipline, courage, or responsibility, whereas Daoist language became salient when continued striving was experienced as excessive, emotionally costly, or dominated by destructive comparison. One participant summarized the complementary functions of the two repertoires:

*Confucianism gave me direction, and Daoism helped my heart settle down.*

The distinction became more explicit when participants linked different responses to different conditions:

*When I was avoiding work, I needed effort; when I was exhausted, I needed to know when to stop.*

This account illustrates why simple co-occurrence was insufficient for coding Situational Switching. The participant did not merely endorse both traditions. Instead, two different situations, avoidance and exhaustion were interpreted as calling for different responses. Striving became appropriate when withdrawal threatened purposeful action, whereas release became appropriate when continued effort risked becoming excessive. What mattered analytically was therefore not the simultaneous presence of two cultural traditions, but the participant’s narrated judgment about which position fitted a particular demand.

Similar movement appeared in participants’ descriptions of culturally available self-positions. Across the narratives, participants could position themselves as persevering learners, self-cultivating future teachers, non-contending peers, or prospective teachers who recognized limits. These positions were neither presented as mutually exclusive nor necessarily stable. Rather, the reflective accounts showed how different culturally storied positions could become available for interpreting different forms of academic and early professional difficulty. As one participant put it:

*One pushes me to stand up; the other reminds me not to lose myself while standing up.*

The statement captures an important distinction between direction and calibration. Confucian striving could provide a reason to continue, while Daoist release could qualify how that continuation should occur. Participants therefore did not always resolve tension by choosing one repertoire over the other. In some accounts, they preserved commitment while revising its intensity, temporarily stepped back from comparison before re-engaging, or interpreted recognition of limits as compatible with rather than opposed to responsibility.

This pattern gives empirical content to the study’s concept of cultural identity regulation. The concept does not imply that participants consciously operated a stable regulatory strategy or that switching can be inferred beyond the written narratives. Rather, it describes how participants narratively repositioned culturally available resources while considering what kinds of effort, pressure, recovery, and responsibility were appropriate to particular situations. Identity regulation was therefore visible less in allegiance to either Confucian or Daoist ideas than in the relationships participants constructed between them.

The pattern also clarifies the boundary cases identified in the first two themes. Moralized Striving occurred when effort lost proportion and became difficult to suspend, whereas Avoidance occurred when letting go became detached from purposeful action. Situational switching occupied the interpretive space between these possibilities: participants narrated the need to judge when persistence remained meaningful and when restoration became necessary. This movement from selecting a culturally appropriate response toward considering what kind of future professional such judgments might support leads to the final theme, in which responsibility, imagined teacher selves, and personal limits were brought together.

### Sustainable teacher formation: responsibility, future selves, and limits

The final theme brought together the operational codes of Teacher Responsibility/Service, Future Teacher Self, and Sustainable Teacher Formation. Across these narratives, participants frequently moved from interpreting their own academic experiences to imagining how they might respond to pupils, professional demands, and classroom uncertainty in the future. Responsibility was therefore not expressed only as an abstract moral obligation. It was increasingly narrated through possible teacher selves: teachers who would encourage perseverance without reproducing excessive pressure, maintain standards while recognizing student differences, and take professional responsibility without assuming complete control over every outcome.

One participant expressed this orientation in relation to mathematics teaching:

*A good mathematics teacher should teach thinking, not only answers. Students should feel that different paths can also be valuable.*

Here, the Future Teacher Self was articulated through a particular understanding of Teacher Responsibility. Good teaching was imagined not simply as transmitting correct knowledge but as creating conditions in which students could think and follow different paths. Similar accounts described patience, guidance, care, and responsiveness as characteristics participants hoped to carry into future teaching. These statements remain anticipatory rather than evidence of enacted classroom practice, but they show how present experiences were used to construct possible professional orientations.

A particularly important pattern was participants’ attempt to reconcile professional seriousness with limits. The imagined teacher self was neither a relentlessly demanding figure nor one detached from responsibility. Instead, several narratives linked commitment with proportion: perseverance remained valuable, but not when it reproduced fear; responsibility remained important, but not when it required endless self-sacrifice; acceptance could be legitimate, but not when it became an excuse for disengagement. Sustainable Teacher Formation was used to capture this higher-order integration of commitment, responsibility, and limits.

Negative and ambivalent cases were especially important in developing this interpretation. One participant described self-strengthening as initially useful but increasingly problematic when it became a demand never to stop. Repeated academic pressure eventually produced the feeling that rest itself represented moral failure. Daoist language about knowing limits subsequently provided a different way of narrating rest, selectivity, and a slower pace not necessarily as withdrawal, but as potentially compatible with responsible preparation. The case does not demonstrate that Daoist language resolved the participant’s difficulty. Rather, it shows how the meaning of Confucian striving could be recalibrated when brought into relation with limits.

The opposite boundary was visible in a Daoist negative case:

*If I say everything follows nature, sometimes I am only making an excuse for not trying.*

This account prevents release from being interpreted as inherently beneficial. Acceptance became problematic when it no longer remained connected to purposeful action or responsibility. Read together, the two negative cases sharpen the distinction developed across the themes: striving without calibration could become moralized self-demand, while release without responsibility could become avoidance. Sustainable formation was narrated in the space between these two possibilities.

The connection to future teaching made this balance professionally meaningful. One participant concluded:

*Responsibility should have warmth. If I only transfer pressure to students, I have not really taught them.*

This statement brings together Teacher Responsibility/Service, Future Teacher Self, and Sustainable Teacher Formation. The participant retained a moral conception of teaching but qualified what responsible teaching should entail. Responsibility was not rejected; it was reconstructed through care and through awareness of how pressure might be experienced by pupils. In this way, experiences of striving, vulnerability, release, and recovery could become resources for imagining a future professional self.

Across the four themes, sustainable teacher formation therefore emerged not as a demonstrated developmental outcome but as a narrated professional orientation toward commitment with limits. Confucian striving provided culturally available language for perseverance, self-cultivation, and responsibility; Daoist release provided language for non-contention, selectivity, and recognition of limits; situational switching showed how participants could narratively reposition these resources according to changing demands. The final theme brought these processes together around an imagined teacher self that remained committed without equating professionalism with continual self-exhaustion.

Figure 2 summarizes these relationships across the four interpretive themes.

**Figure 2:**
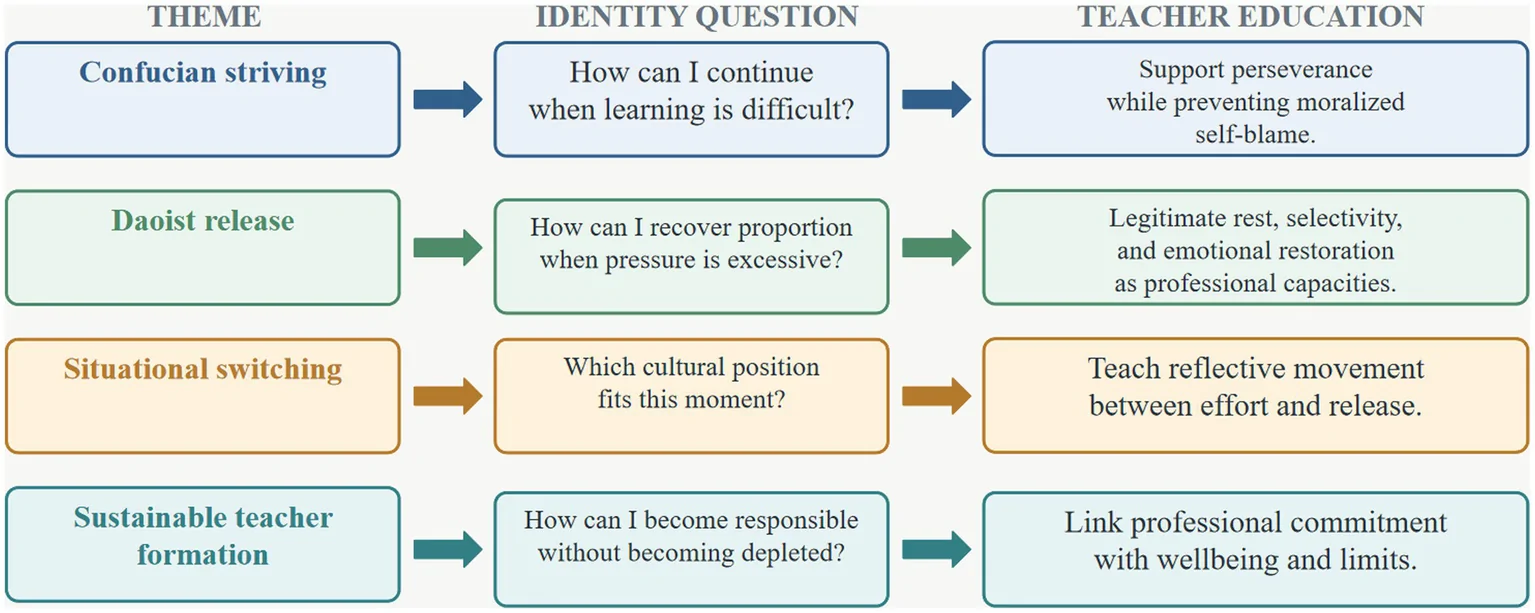
Relationships across the four interpretive themes.

## Discussion

### Repositioning cultural repertoires within teacher identity theory

The principal contribution of this study is to reposition Confucian and Daoist traditions as culturally available resources for pre-service teacher identity work rather than as cultural beliefs that exert uniform effects on professional development. Within this particular teacher education context, participants used these repertoires to interpret academic difficulty, feedback, comparison, emotional strain, teaching preparation, and imagined professional responsibility. Teacher identity scholarship has established that identity is dynamic, relational, and continually reconstructed through biography, professional experience, recognition, and interaction (Akkerman and Meijer, 2011; Beijaard et al., 2004). The present findings add a culturally specific dimension to this account by showing that the language through which professional experiences become meaningful may itself be culturally mediated.

This matters because teacher identity research has given considerable attention to biography, recognition, vulnerability, and institutional positioning, while paying comparatively less attention to the cultural grammar through which these experiences are interpreted (Beauchamp and Thomas, 2009; Kelchtermans, 2009; Rushton et al., 2023). The findings suggest that culturally familiar sayings can function as practical narrative resources through which participants position themselves in relation to difficulty and professional expectation. The same academic setback could be narrated as a demand for perseverance, an occasion for self-cultivation, a source of excessive self-pressure, or a signal that proportion needed to be restored. Culture, from this perspective, does not determine a response; it provides interpretive possibilities through which a response can be made meaningful.

This interpretation also develops the relationship between identity and professional agency. Agency in teaching is increasingly understood not simply as an individual capacity but as situated within professional, relational, and institutional conditions (Buchanan, 2015; Molla and Nolan, 2020). The present findings suggest that agency may also be culturally narrated. Participants did not simply describe whether they persisted or withdrew; they drew on culturally available meanings to distinguish responsible perseverance from excessive striving and restorative release from avoidance. Cultural repertoires therefore appeared less as prescriptions for behavior than as resources for evaluating what kind of response and ultimately what kind of teacher self, seemed appropriate in a particular situation.

### Cultural identity regulation as an analytic heuristic

The concept of cultural identity regulation brings these patterns together. It should not be understood as a new psychological self-regulation construct or as a directly measured internal cognitive process. Rather, it is an analytic heuristic for examining how culturally available meanings enter narrative identity work. Its contribution lies in specifying a process that more general accounts of teacher identity do not always make culturally explicit: participants encounter a dilemma, activate a culturally recognizable interpretive resource, use that resource to authorize or question a response, and connect the resulting interpretation with an imagined professional self.

The four themes make this process visible at different points. Confucian striving provided language for perseverance and self-cultivation, but its moral force could also become vulnerable or self-punitive when persistence was detached from limits. Daoist release provided language for non-contention, selectivity, acceptance, and emotional restoration, but could become avoidance when detached from responsibility. Situational switching showed that participants did not necessarily resolve this tension by endorsing one repertoire over the other. Instead, some narratives differentiated between circumstances requiring renewed effort and those requiring reduction or recovery. Sustainable teacher formation then brought these judgments into relation with future teacherhood, particularly when participants imagined responsibility as requiring both commitment and proportion.

This interpretation refines narrative accounts of professional identity by emphasizing that professional stories may also be cultural stories (McAdams, 2001; Sfard and Prusak, 2005). Participants did not simply recount episodes of studying mathematics or preparing lessons. Within the prompted writing activity, they connected these experiences to culturally available storylines about effort, cultivation, restraint, responsibility, and appropriate conduct. Academic struggle could consequently be narrated as preparation for future professional care, while experiences of pressure could become resources for imagining how not to reproduce similar pressure with future pupils. The important theoretical point is therefore not simply that culture “influences” teacher identity, but that culturally available meanings can provide the narrative content through which particular identity positions become intelligible.

The descriptive corpus layer supports this interpretation while also marking its limits. Recurring language related to learning, effort, self-cultivation, reflection, growth, balance, and future teaching was distributed across the corpus rather than confined to a small number of vivid cases. Yet frequency did not determine meaning. The same language of effort could support a narrative of professional agency in one account and moralized self-demand in another; similarly, acceptance could signify restoration in one narrative and avoidance in another. This distinction reinforces the value of reading corpus-level visibility alongside contextual narrative interpretation (Braun and Clarke, 2021; Byrne, 2022). It also explains why cultural identity regulation is better understood in terms of the situated use and repositioning of repertoires than in terms of stable cultural orientations.

### Cultural identity regulation, wellbeing, and professional sustainability

The findings also contribute, more cautiously, to discussions of teacher wellbeing and resilience. Resilience is increasingly conceptualized as emerging through interactions among personal resources, relationships, and professional contexts rather than as an isolated individual characteristic (Gu and Day, 2007; Mansfield et al., 2016). Similarly, teacher wellbeing is intertwined with identity, emotion, relationships, and the conditions under which teachers experience their professional lives (Dreer, 2023; Squires et al., 2022). The present study adds a cultural meaning-making dimension to this literature: participants used culturally available language to judge when perseverance could be understood as responsible commitment and when stepping back could be understood as a legitimate recognition of limits.

This distinction suggests that wellbeing may enter teacher identity formation not only as an outcome to be protected but also as part of how professional commitment itself is interpreted. Participants’ narratives raised questions about whether they were permitted to rest, reduce comparison, revise expectations, or continue striving while still regarding themselves as serious future teachers. In this sense, emotional restoration and professional responsibility were not necessarily opposites. For some participants, recognizing limits became meaningful precisely because it allowed commitment to be imagined without continual self-exhaustion. This interpretation is consistent with research linking teacher and pre-service teacher wellbeing with identity, emotion regulation, and professional development (Hascher and Waber, 2021; Hui et al., 2024; Squires et al., 2022).

At the same time, the findings do not establish that Confucian or Daoist repertoire use improves wellbeing or produces resilience. The data contain no direct wellbeing outcome measure, nor do they follow participants into practicum or professional teaching. What they reveal is a possible meaning-making pathway through which persistence, limits, recovery, and responsibility became narratively connected. Longitudinal and mixed-method research would be needed to establish whether such interpretations predict or support later wellbeing, resilience, or professional sustainability.

The negative cases are theoretically important here. Confucian striving was not inherently adaptive: its emphasis on self-strengthening could sustain commitment while also intensifying unrealistic expectations and moralized self-blame. Daoist release was similarly ambivalent: non-contention and acceptance could restore proportion, but release could become disengagement when separated from responsibility. The critical process was therefore not the possession of one repertoire or the other, but how its meaning was calibrated within a particular situation. This conditional interpretation is consistent with accounts of teacher identity as negotiated, unstable, and shaped by tensions rather than developing through a simple linear trajectory (Day et al., 2006; Hanna et al., 2022; Nickel and Crosby, 2022).

Seen in this way, sustainable teacher formation is not equivalent to reduced effort or increased comfort. It concerns the possibility of maintaining professional seriousness without converting every difficulty into self-accusation and recognizing limits without converting every difficulty into a reason for withdrawal. The participants’ anticipatory narratives suggest that cultural repertoires can provide language for negotiating this boundary. This is the deeper significance of situational switching: it represents not compromise between two philosophies, but a narrated attempt to preserve responsibility while recalibrating the form that responsibility takes. Such an interpretation extends discussions of wellbeing by connecting emotional calibration with moral purpose and sustainable professional agency (Hui et al., 2024).

Several alternative explanations nevertheless need to remain visible. Because the writing prompt explicitly foregrounded Confucian and Daoist traditions, their prominence cannot be interpreted as evidence of their spontaneous prevalence in participants’ everyday identity work. The accounts may also reflect the positive institutional value attached to diligence, self-cultivation, and teacher responsibility, culturally normative expectations about educational conduct, or social desirability within a classroom writing activity. Moreover, the participants were situated within one normal university and mathematics education context. The present design cannot disentangle these influences. Its contribution is therefore more specific: it demonstrates how culturally available repertoires were mobilized and made meaningful within this prompted corpus, rather than establishing their independent effects on identity or wellbeing.

### Implications for teacher education

The findings suggest several implications for teacher education, although these should be understood as directions for pedagogical design and future evaluation rather than as effects demonstrated by the present study. Reflective practice often asks pre-service teachers what happened during coursework, micro-teaching, or practicum. The present findings suggest the value of an additional question: through what cultural, familial, institutional, or professional language is the experience being interpreted? Reflective approaches are particularly relevant because they connect personal self-understanding with professional identity formation (Rodgers and Scott, 2008).

Teacher education programs need not promote Confucian or Daoist sayings as desirable educational principles. A more productive approach would be to treat cultural repertoires as material for critical reflection. Students could be invited to examine the assumptions embedded in how they narrate failure, feedback, comparison, responsibility, and care. For example, interpreting failed teaching as evidence of personal inadequacy differs from interpreting it as an opportunity for professional learning; similarly, interpreting rest as irresponsibility differs from considering whether temporary reduction might enable more purposeful engagement. Such reflection may make visible the moral expectations embedded in apparently ordinary descriptions of professional experience (Nash et al., 2022).

This approach could be particularly useful in mentoring and practicum contexts, where professional ideals become more consequential. Mentors play an important role in how pre-service teachers interpret professional difficulty and expectations (Izadinia, 2015; Yuan, 2016). Rather than asking only whether a student worked hard enough or performed successfully, mentoring conversations might examine what story the student is constructing about effort, failure, comparison, and care. This could help make visible the point at which a valued ideal such as self-strengthening begins to resemble self-accusation, or when acceptance begins to resemble avoidance. Such questioning is consistent with reflective traditions that treat professional learning as interpretation rather than simple behavioral correction (Bengtsson, 1995; Ketonen and Nieminen, 2023).

One practical possibility would be to invite pre-service teachers to map the sayings, family expectations, professional ideals, or institutional messages they use when interpreting a challenging experience. Reflection could then move beyond identifying the repertoire to asking what response it legitimizes, what kind of teacher self it makes possible, and what risks it may carry. Self-strengthening, for example, may authorize perseverance but may also moralize difficulty; non-contention may loosen destructive comparison but may become avoidance when detached from responsibility. Structured reflection of this kind would be consistent with teacher identity approaches linking self-understanding, vulnerability, and professional learning (Kelchtermans, 2009; Rodgers and Scott, 2008). Its effectiveness, however, would need to be evaluated through intervention or longitudinal research rather than inferred from the present narratives.

### Beyond the Chinese context

The wider relevance of the study lies in the analytic process rather than in the specific cultural traditions examined. The findings do not imply that Confucian or Daoist repertoires should be transferred to other teacher education systems. Instead, they raise a broader question about the culturally available moral languages through which pre-service teachers interpret professional vulnerability. Teacher identity is always situated within particular institutional and cultural environments (Flores and Day, 2006), and different settings may make different repertoires available for interpreting what counts as effort, care, responsibility, failure, or legitimate recovery.

In European or North American contexts, such repertoires might include discourses of reflective professionalism, child-centered care, vocation, accountability, inclusion, or resilience; elsewhere, they may involve religious narratives, family obligations, civic ideals, community responsibility, or other culturally embedded understandings of teaching. This possibility is consistent with research demonstrating the cultural and contextual variability of beliefs and professional identities (Rosenfeld et al., 2022). The transferable proposition is therefore not that teachers everywhere switch between equivalents of Confucianism and Daoism, but that professional identity work may involve selecting, negotiating, and sometimes resisting culturally available moral languages.

Comparative research could test this proposition more directly. Studies across institutions, disciplines, and national contexts could examine which repertoires become available, how they are activated or resisted, and whether their functions change across coursework, practicum, and early-career teaching. Longitudinal designs would be particularly valuable for determining whether the possible teacher selves narrated during preparation are sustained, revised, or abandoned when participants encounter actual classroom responsibility. Such work would extend research showing that teacher identity is responsive to educational context and professional transition (Flores and Day, 2006; Meyer et al., 2023; Ye et al., 2025).

The present study therefore offers cultural identity regulation as a sensitizing analytic heuristic rather than a universal model. Its value lies in directing attention to a question that teacher identity research can sometimes leave implicit: not only how do teachers interpret professional experience?, but also what culturally available languages make particular interpretations of effort, vulnerability, responsibility, and recovery possible? Addressing that question may provide a more culturally grounded account of how professional identities are narrated, negotiated, and imagined across teacher education contexts.

## Limitations

This study has several limitations. The findings are based on prompted reflective narratives from one Chinese normal university and therefore capture retrospective and anticipatory meaning-making rather than identity construction in practice. The mathematics education context and the lack of verified demographic distributions may also limit transferability. Because the prompt explicitly foregrounded Confucian and Daoist traditions, their prominence in the corpus cannot be taken as evidence of their spontaneous role in everyday identity work, and the classroom setting may have encouraged socially desirable responses. The study also cannot determine whether narrated movement between striving and release persists over time or translates into professional practice. Future research should follow pre-service teachers through practicum and early-career teaching, incorporate longitudinal and observational data, and compare institutions, disciplines, and cultural contexts. Further work could also examine how mentors respond when pre-service teachers use cultural repertoires to negotiate pressure, care, responsibility, and professional limits (Sullanmaa et al., 2023).

## Conclusion

This study repositions Confucian and Daoist traditions as culturally available resources for pre-service teacher identity work. Participants used Confucian striving to narrate perseverance, self-cultivation, and responsibility, while Daoist release provided language for non-contention, recognizing limits, and emotional restoration. More importantly, some narratives showed situational movement between these repertoires, suggesting that professional becoming may involve not simply commitment to particular cultural values but judgment about which cultural resources are meaningful under changing demands. The study therefore proposes cultural identity regulation as an analytic heuristic for understanding how culturally available meanings connect academic experience with possible teacher selves. Sustainable teacher formation emerged as an orientation toward commitment with limits, balancing seriousness with proportion, responsibility with restoration, and moral aspiration with humane limits rather than as a demonstrated developmental outcome.

## Data Availability

The dataset can be requested from the corresponding author via email based on ethical review or other reasonable demands.

## Author’s note

Xuefei Lin currently serves as a lecturer at Yangzhou University and is a member of a Shanghai Key Laboratory of Pure Mathematics and Mathematical Practice. He is currently dedicated to evaluating the use of information technology and the dissemination of mathematical culture in vocational schools and high schools.

Qi Tan is a lecturer at Hunan Normal University. Her research interests include mathematics education, educational technology, and teacher education.

Bin Xiong is a professor at East China Normal University and the head coach of the China Mathematical Olympiad Competition. He is committed to the education of gifted students in mathematics and to AI solutions to mathematical problems.
