# Supplementary material for: How do cultural repertoires shape sustainable teacher identity? Confucian striving and Daoist release among Chinese pre-service teachers
Source: Front Psychol. 2026 Oct 15;17:1986498. doi: 10.3389/fpsyg.2026.1986498 (PMC13716601; doi:10.3389/fpsyg.2026.1986498)
Supplement: Supplementary file 1 [file Supplementary_file_1.pdf]

Appendix A. Codebook

Level	Code	Definition	Include when	Exclude when	Anchor example
Confucian Striving	CS1 Confucian Striving	Use of a Confucian-oriented resource to frame sustained effort, discipline, perseverance, or renewed action in response to difficulty.	Effort is presented as a response to setback, avoidance, demanding preparation, or the moral seriousness of learning to teach.	Routine studying with no interpretive function; self-improvement framed mainly as character cultivation (CS2); responsibility toward others without effort (TR).	"Self-strengthening made me divide the large task into today's small task. Only after doing that did I feel that I could continue."
	CS2 Self-Cultivation	Interpretation of difficulty, reflection, learning, or self-correction as material for moral, character, or professional self-development.	The participant explicitly reframes experience as cultivating, correcting, disciplining, or improving the self, including relational self-correction.	Persistence alone; generic growth language without self-evaluative or formative meaning; future teaching aspiration without present self-formation.	"I stopped thinking that difficulty meant I was not suited to mathematics. I began to see difficulty as the place where I should cultivate myself."
	VS Vulnerable / Moralized Striving (negative case)	Striving that becomes endless self-demand, self-blame, moralized pressure, or inability to permit rest.	The participant presents perseverance/self-strengthening as psychologically costly, compulsory, or tied to moral failure when resting.	Ordinary challenge, diligence, or demanding preparation that remains proportionate.	Rest came to feel like "moral failure" when self-strengthening became a demand never to stop.
Daoist Stress	DR1 Daoist Acceptance/	Use of a Daoist-orien	The passage legitimizes stopping,	Simple inactivity;	"Reducing was not

Level	Code	Definition	Include when	Exclude when	Anchor example
Relief / Release	Release	ted resource to loosen excessive control, accept limits or uncertainty, reduce unhelpful effort, or regain proportion.	reducing, accepting, selecting, slowing down, or relinquishing an excessive demand while maintaining meaningful engagement.	giving up without reflective recalibration; disengagement rationalized as naturalness (AV); non-comparison specifically (DR2).	laziness; it was the beginning of understandi ng.”
	DR2 Non-Content ion / Comparison Release	Reframing competition, ranking, or social comparison so that another person’s performance no longer determines self-worth or action.	Explicit reference to ranking, scores, peers, competition, comparison, or non-contention followed by reduced comparison pressure.	General calm or acceptance with no comparison target; collaborative tolerance without competitive comparison.	“Non-conte ntion did not mean giving up. It meant not letting one score decide my worth.”
	DR3 Knowing Limits / Emotional Restoration	Recognition of psychologic al, attentional, or practical limits that permits recovery, emotional space, patience, or sustainable pacing.	The participant identifies overload/exhaustion and responds through rest, emotional settling, selectivity, patience, or a slower pace.	Ordinary rest with no interpretive link to pressure; avoidance of necessary work; simple acceptance of an outcome without restoration.	“When I stopped comparing for a while, I could breathe and study again.”
	AV Avoidance / Maladaptive	Use of release, naturalness,	The participant recognizes that ‘letting go,’	Legitimate rest, reduction of ineffective	“If I say everything follows

Level	Code	Definition	Include when	Exclude when	Anchor example
	Release (negative case)	acceptance, or non-action as a rationale for evading necessary effort or responsibility.	following nature, or accepting limits can become an excuse for not trying or disengaging.	effort, or acceptance that supports later engagement.	nature, sometimes I am only making an excuse for not trying."
Responsibility and Service	TR Teacher Responsibility / Service	Orientation toward responsibility, care, guidance, service, fairness, or moral obligation to pupils/others as part of teacher becoming.	The passage emphasizes what the participant owes to students, peers, community, or the teaching role.	Merely mentioning becoming a teacher; career intention without responsibility; self-improvement that has not been connected to others.	"Before blaming others, I had to ask whether I had really listened, explained clearly, and taken responsibility for my own part."
	FT Future Teacher Self	Explicit projection of present learning, emotion, or cultural interpretation into an imagined future teacher identity or future classroom practice.	The participant states what kind of teacher they hope/expect to be, how they would teach, or how present experience will inform future pedagogy.	Generic references to teaching/major/practicum with no imagined self or pedagogical projection; responsibility in the present only.	"I hope to pass perseverance to my students, but not the pressure that once made me afraid of every mistake."
Identity Regulation	SW Situational Switching	Narrated movement between striving and	The passage explicitly contrasts moments/conditions, changes response	A narrative merely mentions both Confucianism	"When I was avoiding work, I

Level	Code	Definition	Include when	Exclude when	Anchor example
		release (or between culturally storied positions) in response to changing situational demands.	over time, or judges that one situation calls for effort while another calls for stopping, acceptance, or recovery.	and Daoism; two codes appear in separate passages without a stated relation; abstract claim that both traditions are useful.	needed effort; when I was exhausted, I needed to know when to stop.”
	SF Sustainable Teacher Formation	Integration of professional commitment with limits, restoration, proportion, or flexible judgment, producing an imagined form of teacherhood that can be maintained without chronic self-sacrifice.	The passage connects responsibility/teacher commitment with boundaries, wellbeing, proportion, flexibility, or avoidance of transmitting harmful pressure.	Wellbeing alone; rest alone; generic teacher aspiration; simple presence of both Confucian and Daoist ideas without integration.	“Responsibility should have warmth. If I only transfer pressure to students, I have not really taught them.”
